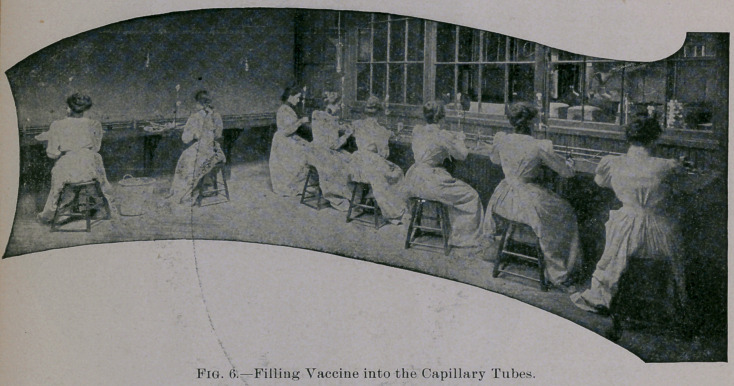# Jenner’s Immortal Discovery

**Published:** 1899-12

**Authors:** 


### Abstracts and Selections.

### JENNER'S IMMORTAL DISCOVERY.

### The Extinction of Smallpox Well-Nigh Accomplished by Vaccine Virus—Some Startling Figures—Delicate, Precise, and Painstaking Methods of Producing and Testing the Lymph—Recent Improvements in Manufacture—How Disease Germs are Excluded.

The vivid descriptions of smallpox epidemics in the pages of the great historians ought to teach us modern mortals what the loathsome disease must have meant in horror and dread to all mankind before the efficacy of vaccination became generally acknowledged.

Even more impressive than the classical pictures of the historians is the evidence presented by the statistics in which are crystallized the experience of entire nations. A calamitous smallpox epidemic raged in Germany during 1870-1, carrying off 143,000 victims in a population of 50,000,000, and in 1874 a law was enacted making vaccination obligatory in the first year of life and revaccination also obligatory at the tenth year.

In consequence of this law smallpox has been so successfully stamped out in Germany that the annual loss of life from this disease is only 116.

Similar figures are afforded by every civilized country, and the lesson they teach is reënforced by the disastrous experience of many careless communities which have temporarily neglected to perform systematic vaccination among the people. The city of Montreal can bear sorrowful witness, from its epidemic in 1885, and the English city of Gloucester, from its outbreak of smallpox in 1896, to the appalling evil which is likely to follow concessions made to anti-vaccination sentiment.

OPPONENTS OF VACCINATION.

The principal stock in trade of those who oppose vaccination is borrowed from the ancient and discarded method of "arm to arm" inoculation, syphillis and possibly other diseases being thus communicated from child to child. In the vehement objections to animal vaccine the tubercular germ has been the great bugaboo. But our

FIG. 2.—"Ready to Invert."—Aseptic Instrument and Dressing Case on the Right.

methods of selecting cattle and our use of glycerin to kill any possible germs in the vaccine exclude that danger perfectly.

But to these unfounded and childish grounds of opposition must be added others of more weight and truth. Not without reason have the antivaccinationists protested against the ulcerations, inflammations, abscesses, and sloughings with which vaccinators have been only too familiar in the past, thanks to the general use of germ-infected "points." The cry of reprobation against these things is not to be silenced by calling people cranks when our best authorities and warmest advocates of vaccination tell us that the old-fashioned "points" fairly *swarm* with disease germs.

THE PROBLEM FAIRLY STATED.

When we decided to place vaccine on the market under our label, we felt that *at any cost* our product must be *the best product obtainable*, otherwise we had better keep out of the vaccine business. And now we purpose to sketch very briefly and rapidly the means we use to preserve our vaccine from infection—measures of asepsis and antisepsis which could hardly be made more minute and painstaking in a modern hospital where patients are prepared for dangerous operations.

THE ANIMAL.

We use only the healthy heifer about eighteen months old. The

FIG. 3.—One Row of Inoculated Heifers in Propagating Room.

animal is first carefully examined by our veterinarian, Dr. E. A. Grange (formerly Michigan State Veterinarian), for any evidence of disease, external or internal. A ringworm on a heifer is enough to condemn it. *The tuberculin test is applied in every case*, and any heifer which exhibits a suspicious rise of temperature is rejected.

INOCULATING THE HEIFERS.

When the animal is finally pronounced to be in perfect health, it is scrubbed from head to foot and taken into the Operating Room—a large, high chamber, with cement floor and varnished walls susceptible of ready cleansing and disinfection. Here, with the aid of a convenient apparatus (see Fig. 4), the heifer is placed on its back; the abdominal surface is thoroughly lathered, washed, and *shaved*, and is then scrubbed once more with sterilized water; it is then washed thoroughly with a disinfectant solution; and after a final washing with sterilized water, the abdomen is ready for

SCARIFICATION.

This is performed quickly with sterilized instruments. The "seed" vaccine is applied, rubbed in thoroughly, and permitted to dry. The "field" of operation is then covered with an *aseptic and impenetrable*

FIG. 4.—Collecting the Vaccine.

cement which effectually excludes germs. Over the cement we place a layer of absorbent cotton, and over the cotton a protective bandage.

(Other manufacturers of vaccine merely cleanse the abdominal surface. So far as we are aware, they do not use a disinfectant, nor do they cover the "field" with anything, simply allowing nature to form a scab.)

The heifers are now ready for the

PROPAGATING ROOM.

Figure 3 shows one row of iron stalls. Here the inoculated animals are kept for about five days. Men are on hand constantly to collect feces, etc., all excreta being removed from the room immediately.

COLLECTING THE VIRUS.

After about five days the heifer returns to the Operating Room. The hoofs are carefully cleaned, and the various cleansing operations described above as preliminaries to inoculation are now *repeated.*

FIG. 5—Grinding Vaccine with Glycerin.—Emulsifying Apparatus to the Left.

The dressings are removed; the whole field of operation is cleansed with sterilized water and disinfectant solution; and the external scab is removed *and destroyed.*

The pulp of the vaccine vesicles with exuding serum is now carefully collected with sterilized spoon curettes and placed in sterilized containers filled with glycerin.

MANIPULATION OF THE LYMPH.

The vaccine is now brought to our Biological Laboratory, and is run through sterilized grinders until a *homogeneous mixture* is obtained. The requisite amount of diluent is added, and the mixture is shaken for several hours in a specially devised shaking apparatus in order to make a perfect emulsion.

SEARCHING SCRUTINY OF THE FINISHED PRODUCT.

The vaccine is now examined *bacteriologically and physiologically.* Every single parcel of our vaccine is tested on heifers before we permit it to go out on the market under our label. And in the great majority of cases our vaccine is tested for activity on children as well.

FIG. 6.—Filling Vaccine into the Capillary Tubes.

If our test requirements are fulfilled, it is filled by skilled operatives into sterilized tubes, in an Aseptic Room especially designed for the purpose. Each tube is examined to satisfy us that both ends are absolutely closed.

PROPER STORAGE OF VACCINE STOCK.

The sealed tubes are at once placed in a refrigerator and kept there until needed for orders. We aim to send out only strictly fresh vaccine, and our stock is changed every week. The proper storage of vaccine by our patrons is of the utmost importance. Vaccine is a most delicate and perishable product. *Keep it in a cool, dark place* (best of all, in a refrigerator), and by all means avoid exposing it for any length of time to a temperature above 70° F. During warm summer weather vaccine deteriorates very fast.

THE "SEED" VACCINE.

This is, of course, the corner-stone of our process; and we insure its activity by our stringently careful preservation and by frequent tests.

WHAT THE TRUE VACCINA VESICLE LOOKS LIKE.

Pray, remember that the so-called vesicle is the *only reliable indication* that the vaccine has "taken." *There is absolutely no other proof for or against the vaccine.* A hole in a man's arm half an inch deep—a scar two inches long—proves nothing (except that infection more or less serious has occurred), and neither one affords any guarantee of protection against smallpox. On the other hand, Jenner himself declared that a full measure of such protection is imparted by a single vesicle. The latter varies in size, but is usually *umbilicated* or depressed in the center. At one stage in its growth the vesicle is filled with pearly-gray matter. Often it is small and escapes observation. *Pure vaccine ought to produce only a mild reaction.* Violent symptoms, local or constitutional, point to infection, either from the vaccine itself or through careless exposure of the wounded arm *after* vaccination.—From advance sheets, *"Therapeutic Notes,"* kindly furnished by Parke, Davis & Co.

## Figures and Tables

**Fig. 2. f1:**
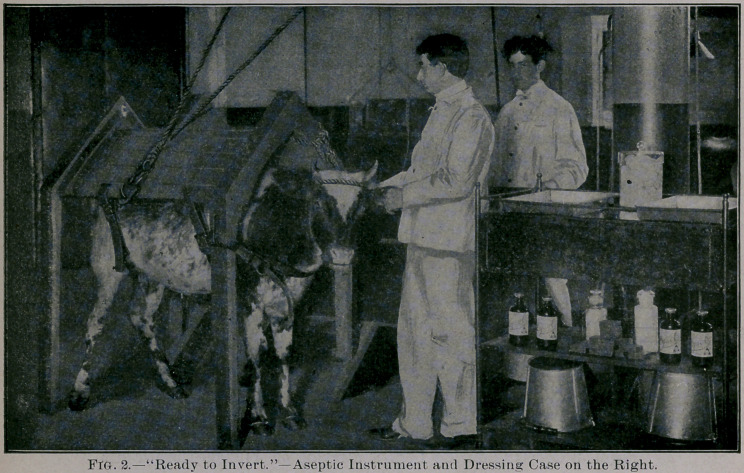


**Fig. 3. f2:**
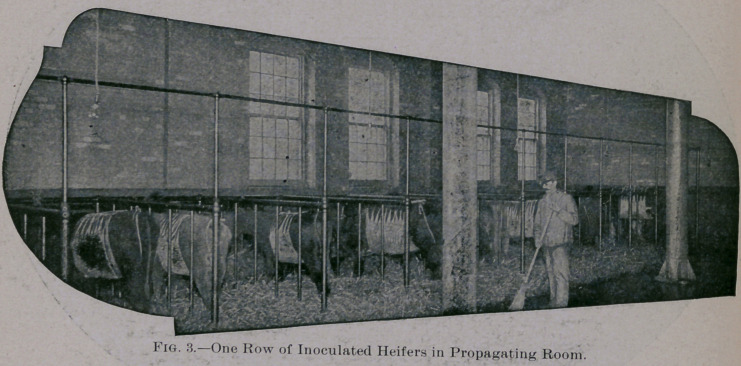


**Fig. 4. f3:**
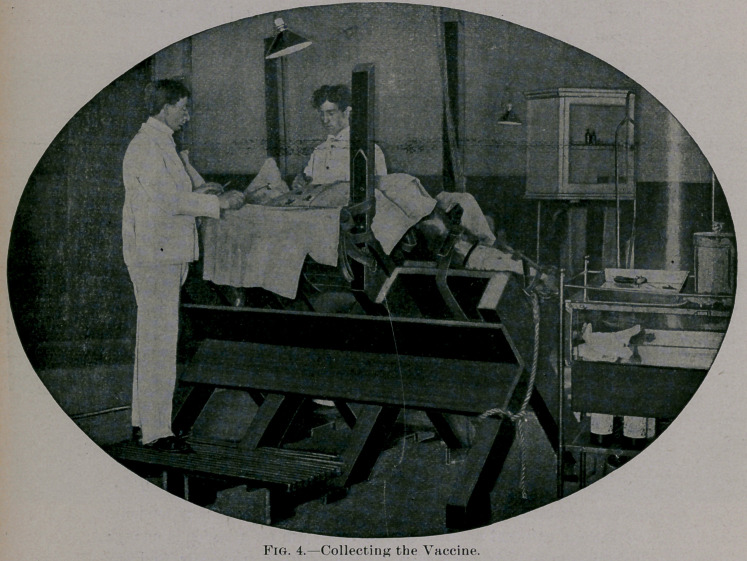


**Fig. 5. f4:**
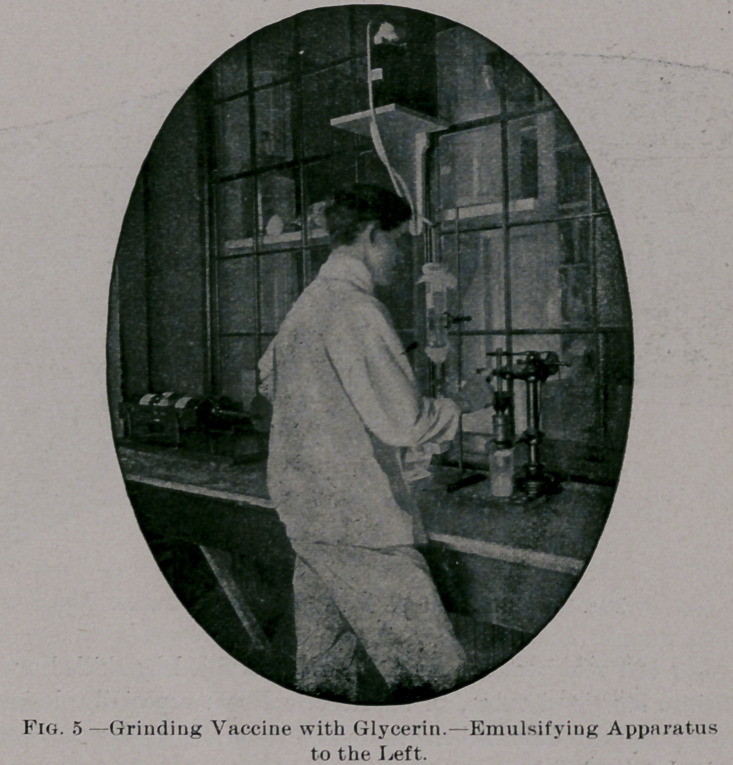


**Fig. 6. f5:**